# Development of Genomic Resources for Pacific Herring through Targeted Transcriptome Pyrosequencing

**DOI:** 10.1371/journal.pone.0030908

**Published:** 2012-02-27

**Authors:** Steven B. Roberts, Lorenz Hauser, Lisa W. Seeb, James E. Seeb

**Affiliations:** School of Aquatic and Fishery Sciences, University of Washington, Seattle, Washington, United States of America; American University in Cairo, Egypt

## Abstract

Pacific herring (*Clupea pallasii*) support commercially and culturally important fisheries but have experienced significant additional pressure from a variety of anthropogenic and environmental sources. In order to provide genomic resources to facilitate organismal and population level research, high-throughput pyrosequencing (Roche 454) was carried out on transcriptome libraries from liver and testes samples taken in Prince William Sound, the Bering Sea, and the Gulf of Alaska. Over 40,000 contigs were identified with an average length of 728 bp. We describe an annotated transcriptome as well as a workflow for single nucleotide polymorphism (SNP) discovery and validation. A subset of 96 candidate SNPs chosen from 10,933 potential SNPs, were tested using a combination of Sanger sequencing and high-resolution melt-curve analysis. Five SNPs supported between-ocean-basin differentiation, while one SNP associated with immune function provided high differentiation between Prince William Sound and Kodiak Island within the Gulf of Alaska. These genomic resources provide a basis for environmental physiology studies and opportunities for marker development and subsequent population structure analysis.

## Introduction

Many commercially exploited species face ecological and anthropogenic pressures in addition to fisheries, such as pollution, emerging diseases and climate change. Although demographic effects of such pressures are difficult to quantify, they are likely to affect both ecosystem structure and economic returns of dependent fisheries. Studies on the genetic and organismal effects of these pressures may provide insights into the phenotypic flexibility and the scope for adaptation that may allow resilience and resurgence of exploited populations. For example, Pacific herring (*Clupea pallasii*) in Prince William Sound (PWS) have collapsed after the *Exxon Valdez* oil spill in 1989, resulting in a closure of commercial and traditional fisheries. Despite two decades of research and extensive restoration efforts, Pacific herring is one of only two resources still classified as ‘not recovered’ [1]. Although herring spawning populations were large in 1989, the recruiting cohort was one of the weakest on record, and by 1993, the spawning population was reduced to about a quarter of its previous size [1]. Even now, the population has not recovered [2], and remains well below the recovery aim of 43,000 tons [1]. Because of the central position of herring in the marine food web and its importance as a commercially exploited species, such low biomass may affect the entire ecosystem as well local fishing communities.

The causes for the initial collapse of PWS herring are not well understood, and even the exact timeline of the collapse is under dispute [2], [3]. Nevertheless, exposure to oil pollution [4], disease [5], [6], predation/competition [7] and changes in the physical oceanography, or any combination of these factors, are potential culprits. Although it may not be possible to reconstruct the exact causes of the collapse, it is important to identify factors limiting or preventing recovery [1]. The combination of new molecular technologies allowing the sequencing of the entire expressed genome (transcriptome) of non-model species and novel computational approaches provide the opportunity for efficiently addressing potential causes underlying the lack of Prince William Sound herring recovery through the development of genomic resources. New sequencing technologies have greatly reduced the costs required for genomic resource development, though there are still challenges faced when working with non-model organisms [8], [9]. Short sequence read lengths and large quantities of data have to be analysed *de novo*, without the assistance of a reference genome that would be available for species such as humans, mice, and zebrafish. Following the initial steps of assembly and annotation, putative genetic markers can, however, be more easily compared to older sequencing technologies given the large quantity of sequencing reads. The large number of putative markers that can be identified greatly increases the potential to identify self-recruiting populations, even if the populations are large and connected by relatively high migration rates.

We report the sequencing of the herring hepatic and testicular transcriptome in order to provide a more comprehensive set of genomic resources for Pacific herring for population structure analysis and environmental physiology studies. This represents the first large scale sequencing efforts for a member of the teleostean order Clupeiformes. An annotated transcriptome is described, as well as a workflow for SNP discovery and validation. Furthermore, we provide preliminary analysis on population structure at select genes and compare patterns of diversity and differentiation at loci developed from this effort to allozyme, microsatellite, and mitochondrial DNA markers screened in the same populations.

## Results

### Sequence Assembly and Annotation

**Figure 1:**
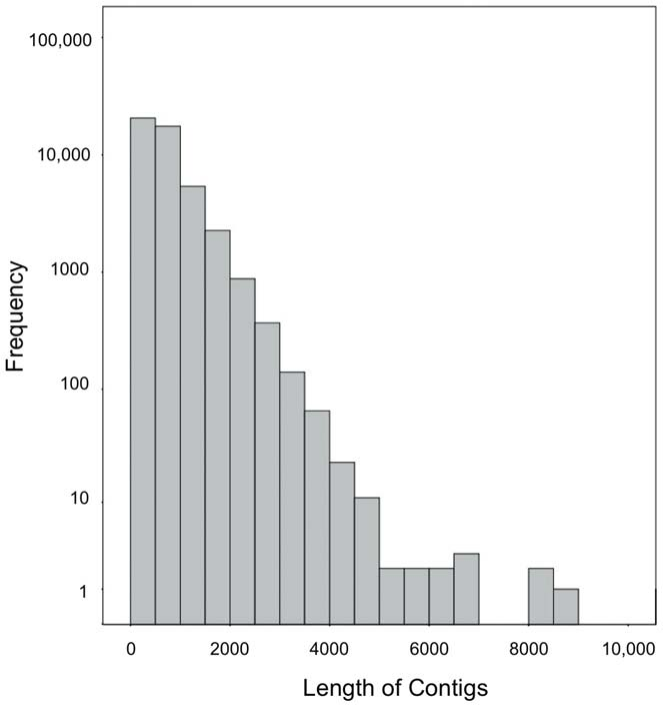
Histogram of contig sequence length. Contig sequences were generated from *de novo* assembly of both libraries (n = 42,953) and average length is 728 bp. Note logarithmic scale for frequency axis.

**Figure 2:**
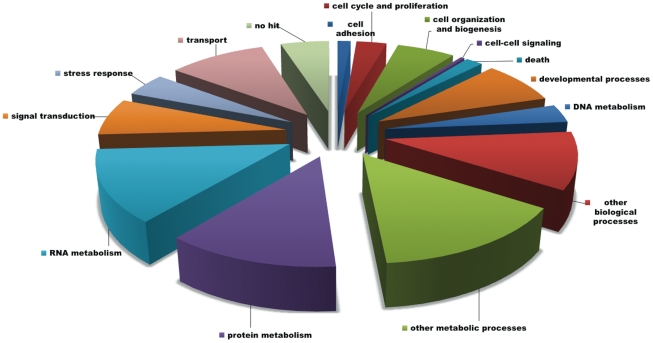
Functional categorization of contig sequences. Contig sequences were generated from *de novo* assembly of both libraries (n = 42,953) and annotations performed using BLASTx with Swiss-Prot and Gene Ontology databases.

In total, 2,117,781 raw sequencing reads were generated with an average length of 254 bp (Table 1). All data was submitted to NBCI's Short Read Archive under accession number SRX022719. After quality trimming, 96% of the data was retained for a total of 530 Mb of sequencing data. Quality trimmed reads from the liver and testes libraries were *de novo* assembled separately to generate 34,300 and 31,545 contiguous sequences (contigs), respectively [10], [11]. *De novo* assembly of all data resulted in 42,953 sequences with an average length of 728 bp. A majority, 81% of the contigs, were between 100 and 1000 bp in length (Figure 1). A majority of the contigs were classified as involved in protein metabolism, RNA metabolism, or other metabolic processes (Figure 2).

**Table 1:** Characteristics of Pacific herring hepatic and testicular transcriptome sequencing.

	Sequences (n)	Average Length
*Raw Sequencing Reads*		
Liver Library	1,195,565	278
Testes Library	982,216	233
Both Libraries	2,177,781	254
*Quality Trimmed Reads*		
Liver Library	1,109,404	284
Testes Library	837,401	257
Both Libraries	1,946,805	272
*Contigs*		
Liver Library	34,300	625
Testes Library	31,545	646
Both Libraries	42,953	728
*Singletons*		
Liver Library	749,929	284
Testes Library	315,852	266
Both Libraries	778,383	259

### Library Comparison

**Figure 3:**
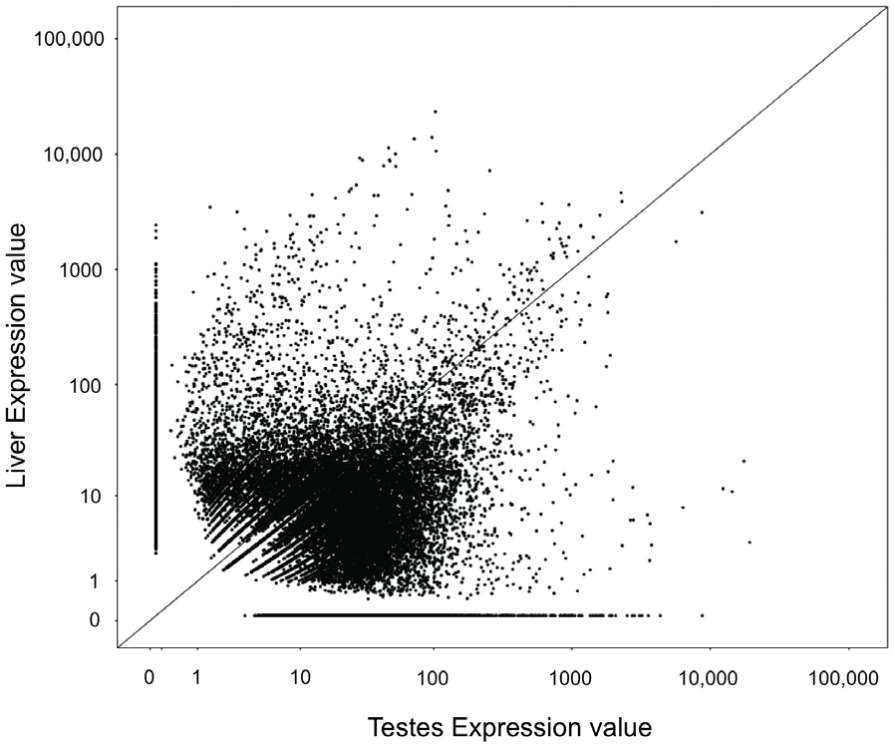
Relative expression (RPKM) of 454 sequenced transcriptome across liver and gonad tissue. Diagonal line represents equal expression in both tissue types. Note both axes are on the logarithmic scale.

To investigate the relative contribution of each library to the rate of gene discovery, RNA-seq analysis was performed. The testes tissue library included 15,401 features expressed at a higher level (>4-fold) with 13,379 features expressed at a higher level in the liver library (Figure 3). A large number of features, 8,346 in testes library and 11,185 in liver library, were expressed in only a single library.

**Figure 4:**
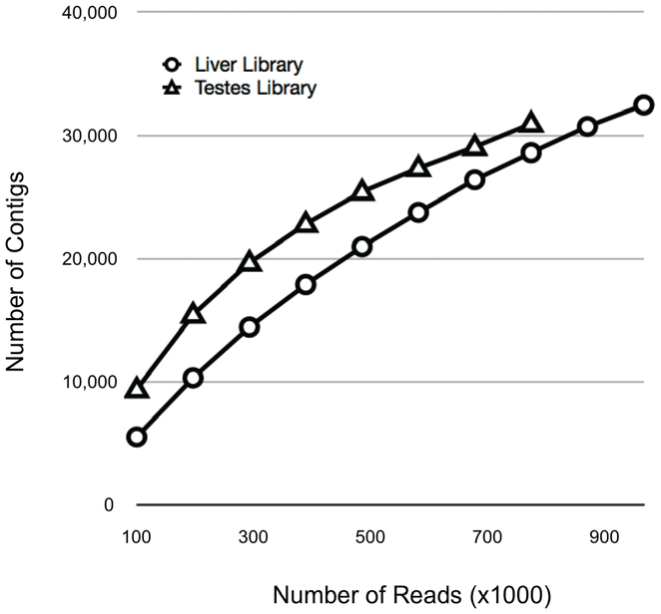
Rarefaction analysis of quality trimmed reads from each library. Rarefaction analysis was used to determine level of contig discovery relative to sequencing effort. Reads were sequentially sampled in 1×10^5^ sequence read increments and *de novo* assembled.

The number of contigs per number of reads was lower in the liver library compared to the testes library. The percentage of reads that generated the contigs varied across libraries with the percentage ranging from 62% to 69% for the liver library and from 52% to 62% in the testes library. When sequencing effort was reduced *in silico* by approximately 50% (500,000 reads/library) 20,966 and 25,416 contigs were generated from the liver and testes libraries, respectively (Figure 4).

### SNP Discovery

SNP detection analysis revealed 10,933 potential SNPs in the combined herring transcriptome. A majority of the SNPs (60%) were transitions. A/T and C/G transversions were each present in 9% of the candidate SNPs while the G/T and A/C substitutions constituted 11% of the polymorphisms. Average coverage of putative SNPs was 16.6 (SD = 46.8), with 95% of the SNPs having coverage less than 25×.

Distinguishing synonymous and non-synonymous SNPs from high-throughput sequence data in species without sequenced genomes can be a challenge. Over both libraries, 161,059 potential open reading frames were identified. SNP detection using these open reading frames to map all quality trimmed reads revealed 4448 putative SNPs. Of those SNPs, 1610 resulted in a predicted amino acid substitution. After removing sequences with e-values greater than 1.0E-10 (Swiss-Prot database) and less than 10× coverage, 257 non-synonymous SNPs and 722 synonymous candidates remained (dn/ds = 0.356).

### SNP Validation and Population Screening

Fifty candidate SNPs did not pass the initial primer testing; many of these were likely true SNPS adjacent to intron/exon boundaries and would not amplify. Sanger sequencing confirmed the presence of one polymorphism in 14 templates and two or more polymorphisms in 16 templates. The 14 templates with a single polymorphism were used for HRMA. Additional Sanger sequencing demonstrated the presence of more than one polymorphism in four of these sequences in other populations (Table 2). The 14 templates were originally identified based on functional annotation of the respective transcript. Some loci did not have significant BLAST hits when the targeted genomic region was examined, however several loci are likely associated with genes involved in immune and xenobiotic response (Table 3).

**Table 2:** Loci selected for HRMA and associated primers.

Loci ID	Primer 1	Primer 2	SNP Position	SNP
Cpa_28881	TCGTTCTGATTGGCTTACCC	GTTGGGGCTTGCCTAAAAAT	73, 93, 128	G/T, C/T, C/G
Cpa_RG9MTD2	CTGCCACAGTGTGTGTACCAT	CTCTCTGCCAGTGATGCTGA	84, 109	C/T, C/G
Cpa_ABCG5	CCACCGTCCAGTAGAGGAAT	TTTCCTGCACTCAGGGCTAT	75, 90, 121	G/T, G/T, A/G
Cpa_SOX11	TTGCTACAAAACGCAGATGG	GTGAATGGGTCCCACATAGC	90	A/C
Cpa_24210	TTGGACAAGCGTGTTGTGTT	GTAAGGAATGCCCACGTCTG	70	A/C
Cpa_28757	AAGGATGCCAACAGCACTCT	CCCTCAGAGGTTTCATGGTG	64, 77	A/G, A/G
Cpa_CYP2J5	TGTCTTTGGTGGCACTTCTG	GAGGAGATTGACCGTGTGGT	46	A/G
Cpa_UGT2A	CTCCTGAACTCCGTTCGTTC	AGGTCATCTGGAGGCATCTG	85	C/T
Cpa_11680	TCTTCGCACAATGACCACTC	GGCTTTAGCAATTAGCTGCAT	87	A/C
Cpa_11961	TCATCAGGCGTTGACAAAGA	GTCGACTGCTTGAGGAGACC	69	G/T
Cpa_PM20D1	GTGACTGTGTTGGGCATGAG	CCAACCCTGATGTCAGTTCC	49	C/T
Cpa_11785	CTGAGGGCTCTTTGGCTTTA	GGTTAAGAGGGCCGGTAAAA	63	A/G
Cpa_UPF0669	CACTTCGAGGACGATGATGA	GGCTGCTCATGTGTAGGATG	65	A/G
Cpa_APOB	TTGCAGTACCCTCAGTGGTG	AGGTGTCTGCCAAGGTCAAC	60	C/T

**Table 3:** Annotation of selected loci based top BLAST hit and GO ontology.

Loci ID	Genomic BLAST Hit	Accession #	e-value	Gene Function
Cpa_28881	no hit			
Cpa_RG9MTD2	RNA methyltransferase domain-containing protein 2 (Human)	Q8TBZ6	9.55E-18	methyltransferase activity
Cpa_ABCG5	ATP-binding cassette sub-family G member 5 (Human)	Q9H222	9.57E-10	cholesterol homeostasis
Cpa_SOX11	Transcription factor Sox-11 (*Salmo salar*)	NM_001173797	2.00E-04	regulation of transcription
Cpa_24210	no hit			
Cpa_28757	no hit			
Cpa_CYP2J5	Cytochrome P450 2J5 (mouse)	O54749	1.34E-11	oxidation reduction
Cpa_UGT2A	UDP-glucuronosyltransferase 2A2 (Human)	Q9Y4X1	2.88E-14	sensory perception of smell
Cpa_11680	no hit			
Cpa_11961	no hit			
Cpa_PM20D1	Probable carboxypeptidase PM20D1 (zebrafish)	Q08BB2	1.62E-12	metal ion binding
Cpa_11785	no hit			
Cpa_UPF0669	UPF0669 protein C6orf120 homolog (zebrafish)	Q6NZZ3	6.53E-14	
Cpa_APOB	Apolipoprotein B-100 (human)	P04114	9.55E-18	response to virus

**Figure 5:**
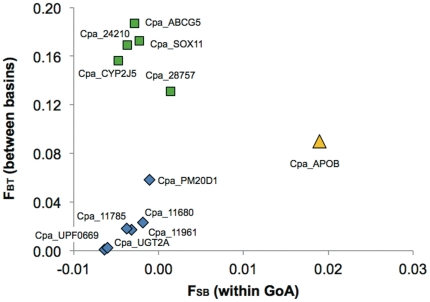
Variance components from a locus-by-locus AMOVA. Five loci (green) showed differentiation between the two ocean basins (high *F_BT_*), while a single locus (yellow) significantly differentiated between Prince William Sound and Kodiak Island within the Gulf of Alaska (high *F_SB_*, yellow).

HRMA showed that eight of the fourteen tested loci conformed to HWE in all three samples (Table 4). Five loci deviated significantly from HWE in one sample (two loci because of heterozygote excess and three because of heterozygote deficiency). One locus deviated significantly from HWE in two samples (both heterozygote deficiency). Four loci deviated from HWE in Togiak herring, two loci in Prince William Sound and one locus in Kodiak Island fish. Average heterozygosity was lower in the Bering Sea (*H_e_* = 0.221) than the Gulf of Alaska (*H_e_* = 0.339) (Table 5). A hierarchical AMOVA showed that 8.7% of the variation was due to differences between populations, and most of that differentiation was due to differences between the Bering Sea (*i.e.* Togiak) and the Gulf of Alaska (Table 6). Eight of the 14 loci showed significant differentiation among populations before (P< = 0.034) and seven after Bonferroni correction (P< = 0.001). Differentiation between ocean basins was higher than within Bering Sea or Gulf of Alaska (Figure 5), but because of the small number of samples and the low power of permutation tests resampling entire collections between groups as carried out in Arlequin, that differentiation between ocean basins was not significant at any locus. However, one locus associated with virus response showed significant differentiation (P = 0.042) between PWS and Kodiak within the Gulf of Alaska (Figure 5).

**Table 4:** Minor allele frequency, expected heterozygosity, and inbreeding coefficient (*F_IS_*) for select loci in three Pacific herring populations.

	Minor Allele Frequency			Expected Heterozygosity			Inbreeding Coefficient (FIS)		
Loci ID	Kodiak	PWS	Togiak	Kodiak	PWS	Togiak	Kodiak	PWS	Togiak
Cpa_28881	0.29	-	0.23	0.42	NA	0.36	−0.05	NA	−0.12
Cpa_RG9MTD2	0.33	-	0.21	0.45	NA	0.34	0.17	NA	**0.47**
Cpa_ABCG5	0.26	0.23	0.01	0.39	0.35	0.01	−0.04	−0.17	0
Cpa_SOX11	0.22	0.25	0.01	0.34	0.37	0.01	−0.08	0.15	0
Cpa_24210	0.25	0.27	0.02	0.37	0.4	0.04	−0.15	−0.15	**0.49**
Cpa_28757	0.26	0.22	0.03	0.39	0.34	0.06	−0.07	−0.08	0.32
Cpa_CYP2J5	0.13	0.14	0.38	0.23	0.25	0.47	**0.43**	**0.55**	0.16
Cpa_UGT2A	0.23	0.25	0.23	0.36	0.37	0.36	−0.21	−0.2	**−0.24**
Cpa_11680	0.32	0.28	0.20	0.44	0.41	0.32	−0.09	0.09	0.19
Cpa_11961	0.31	0.28	0.20	0.43	0.4	0.33	−0.05	−0.09	**0.38**
Cpa_PM20D1	0.28	0.25	0.12	0.41	0.37	0.21	−0.13	−0.15	−0.02
Cpa_11785	0.01	M	0.02	0.01	0	0.04	0	NA	−0.02
Cpa_UPF0669	0.20	0.19	0.17	0.32	0.31	0.28	0.11	−0.16	−0.05
Cpa_APOB	0.29	0.20	0.06	0.41	0.32	0.11	−0.18	**−0.25**	0.14

Numbers in bold represent values that deviated significantly from HWE.

**Table 5:** Unbiased heterozygosity of four genetic markers from Pacific herring; haplotype diversity was used for mtDNA.

	Bering Sea	Gulf of Alaska	Ratio^a^	
**Allozymes**	0.073	0.098	1.34	[22]
**Microsatellites**	0.851	0.905	1.06	[21]
**MtDNA**	0.778	0.883	1.13	[26]
**SNPs**	0.211	0.339	1.61	This study

a the ratio between the two values (Gulf of Alaska/Bering Sea).

**Table 6:** Locus by locus AMOVA of SNPS compared to allozyme, microsatellite and mtDNA.

F component		Allozyme^a^	*F_ST_* Microsat^a^	*R_ST_* Microsat^a^	MtDNA^a^	SNPs
**within basin**	FSB	0.003	0.01	0.03	0.013	−0.001
**Between basins**	FBT	0.241	0.023	0.209	0.169	0.087
**Total**	FST	0.244	0.033	0.233	0.178	0.086

a **From** [**26**] **.**

## Discussion

The large-scale sequencing effort characterized here was carried out to provide a foundation of such genomic information to assist in the research on the biology, ecology, and population genetics of herring. Prior to the completion of our sequencing project, there were less than 1000 publically available nucleotide sequences for the Pacific herring. Furthermore, sequence information from any species of the teleostean order Clupeiformes was limited, and zebrafish (*Danio rerio*) was the most taxonomically similar species with significant genomic resources. Here, we provide over 2 million nucleotide reads from the Pacific herring transcriptome. From these data, we were able to generate over 40,000 contigs with a 10× average coverage depth, identify thousands of putative SNPs, and demonstrate realistic levels of population diversity and differentiation in a small subset of these SNPs.

### Pyrosequencing and non-model species

Pyrosequencing using the 454 platform on non-model organisms is increasingly proven to be an effective and efficient means to provide large scale transcriptomic information. Given the dynamic nature of gene expression, advances in technology and the variety of analytical techniques available make it difficult to directly compare studies, however generally our results are similar to other sequencing efforts. One of the initial applications of 454 pyrosequencing on non-model organisms was carried out on the butterfly, *Melitaea cinxia* [12]. More recently, this platform has been used to provide resources for aquatic organisms of ecological importance. In the lake sturgeon (*Acipenser fulvescens*), 47,060 reads were produced and assembled into 1831 contigs [13]. In chum salmon, two individual fish testes were sequenced and combined, resulting in 1.9 million reads and 118,546 contigs [8]. In both of these efforts, novel SNPs were characterized. In addition to using 454 pyrosequencing for gene discovery and SNP development, the platform provides the opportunity for large-scale expression analysis (RNA-Seq) in organisms of ecological importance. For example, in the lake trout (*Salvelinus namaycush*), 425,821 quality-trimmed reads from liver tissue were assembled into 2276 contigs that were then used for comparative transcriptomic analysis of two lake trout ecotypes [14]. In the studies listed above, methodologies other than pyrosequencing were employed for SNP validation (e.g. HRMA analysis and Sanger sequencing) and RNA-Seq analysis (e.g. quantitative reverse transcription PCR). Likewise, we present the use of HRMA and Sanger sequencing for the validation of SNPs in the Pacific herring. As sequence costs continue to decrease, it is likely that pyrosequencing and other sequencing technologies will be increasingly used for SNP validation and comprehensive RNA-Seq analysis in non-model species.

### Rarefaction Analysis

The number of reads generated per given sequencing effort is relevant, particularly given any financial consideration. In order to characterize the benefit of additional reads to information yield, we examined the number of contigs generated for a given number of quality-trimmed sequence reads (Figure 4). This type of rarefaction analysis is similar to the approach taken by Hale et al. [13] to compare 454 library construction approaches for sturgeon transcriptome libraries. For the herring libraries, a decrease in rate of new contig discovery to below 10% of sequence reads was reached when between 500,000 and 700,000 reads were utilized. This would be expected and indicates with additional sequencing the amount of new information gained will decrease. When the same approach was used to compare incremental number of reads from chum salmon [8], the rate of increase of number contigs was higher, as was the total number of contigs (data not shown). This is likely related to genome duplication events present in the salmonid lineage. As described above, the dynamic nature of transcriptomes and laboratory techniques have to be taken into consideration when comparing libraries and planning sequencing effort. However, the use of rarefaction analysis does provide a simple way to characterize transcriptome diversity.

### Tissue-Specific Gene Expression

While RNA-seq analysis was not used in the study to address ecological issues, the analytical techniques were utilized to evaluate expression differences between the two tissues examined, liver and testes. This approach can provide information on the benefit of the multiple tissues as well as information of functional importance. There was a large difference in the expression levels between tissue types, with over 67% of the features differentially expressed in one tissue. Furthermore, 45% of the features were only expressed in a single tissue. This was expected, given the functional difference in gonad and hepatic tissue, and illustrates tissue diversity is useful for discovery of markers at the genomic DNA level from transcriptome sequences.

### Complement System

RNA-seq-based approaches also allow for the identification of genes associated with specific biological function. One group of genes of particular interest in immune physiology is components of the complement system (see [15] for review). The complement system is an important component of both the innate and acquired immune response. Three pathways are involved in the complement response, including the classical, lectin, and alternative pathways. The classical pathway is activated by antigen and antibody complexes, the lectin pathway requires interaction of lectins with sugar moieties on microbes, and the alternative pathway is initiated by the spontaneous activation (hydrolysis) of C3. Complement C3 in herring was identified in the herring liver and most similar to that of the complement C3 homolog in rainbow trout (Table S1). A complement C2 homolog was identified and is important for activation of both the classical and lectin pathways. Factor B is present, and together with complement C2, both found in the MHC III region in mammals [16]. In contrast to complement C2, Factor B is an important component in the alternative pathway. Activation of each pathway can lead to the assembly of the membrane attack complex (MAC). The membrane attack complex forms transmembrane channels in bacterial pathogens causing cell lysis and death. The membrane attach complex is composed of C5b, C6, C7, C8 and C9 components [17]. C5-C9 homologs were all identified in the herring liver transcriptome (Table S1).

### Cytochrome p450 Superfamily

Several members of the Cytochrome p450 enzyme superfamily were identified in this pyrosequencing effort. In the consensus sequences generated from liver library reads, putative members of families CYP1, CYP2, CYP3, and CYP4 were all identified (Table S1). One of the most studied cytochrome p450 enzymes is CYP1A1. This enzyme is involved in phase I xenobiotic metabolism including dioxins, PCBs, and PAHs. CYP1A1 has served as a key biomarker for petroleum product exposure, as it has been shown to be induced by exposure to these compounds in aquatic species [18]. A 1400 bp consensus sequence was identified in the herring liver transcriptome that codes for CYP1A1. CYP1A1 expression is initiated by activation of the Aryl hydrocarbon receptor, which was also partially sequenced in this project. Partial sequence information of genes in this and other pathways of interest will facilitate research on Pacific herring and are of particular interest given the impacts of the oil spill.

### SNP Development

One primary motivation for the high-throughput sequencing effort on Pacific herring was the discovery of SNPs in coding regions that could be used for population studies on selection and local adaptation. Neutral molecular markers, such as allozymes and microsatellites, have provided powerful insights into population structure and demographic history of wild populations, but they cannot detect adaptive genetic variation. Large-scale genome sequencing efforts combined with outlier tests now have the potential to find genes that are important for adaptation in wild populations. Multiple strategies to increase coverage and target potentially selected genes are available, including exome capture [19], restriction enzyme-based selection [20], and transcriptomic sequencing [12]. For Pacific herring we chose to target the transcriptome of biologically relevant tissue, primarily as this approach provides a wealth of protein encoding sequence that can be used in physiological, ecological, and evolutionary studies and may reveal insights into biological and physiological reasons for the lack of recovery. A drawback to this approach is that, ultimately, population genetic markers will be characterized at the genomic DNA level, and the exclusion of introns in the transcriptome can contribute to marker “drop-out” during validation [8]. For instance, 96 putative SNPs were selected for further validation in this study and only 46 primer pairs produced a single PCR product. For those PCR reactions where no band was produced, it is likely that introns either prohibited primer annealing or resulted in a product too large for amplification. Another reason for the drop-out at this stage was non-specific amplification resulting in the presence of multiple amplicons. One way this could be mitigated in future projects would be to use other known species-specific sequence information to exclude primers without optimal specificity.

Another challenge in developing SNPs in non-model organisms is the determination whether a particular SNP will result in an alteration in amino acid. This information is particularly important when there is interest in selective environmental pressure that could be related to physiological responses and distinguish populations. Here, we developed and generalized a workflow that could be used for any species where there is an absence of an annotated genome. Essentially, a combination of open reading frame identification (based on absence of stop codons) and sequence similarity scores were used to identify SNPs that likely result in a predicted amino acid substitution. Based on the workflow described, we estimated that there were 26% non-synonymous SNPs. It should be pointed out we did not validate this prediction and only a fraction of SNPs (979 SNPs) were available for this form of characterization.

### Preliminary population genetic analysis

Most loci conformed to HWE, even before Bonferroni correction, and significant deviations from HWE showed no clear concentration towards specific loci. However, the Bering Sea population (Togiak) had more loci that were out of HWE than populations from the Gulf of Alaska, which may be due to ascertainment bias or selection, but this needs further investigation. Ascertainment bias seems possible because even though sequences were obtained from a group of fish that included Bering Sea fish, three quarters of the fish originated from the Gulf of Alaska. It may be that in Bering Sea fish, these SNPs included a non-amplifying null allele more commonly known from microsatellite, though this would need to be confirmed by additional sequencing of homozygotes. Four templates had more than a single SNP, but three of the four showed no significant deviation from HWE in any of the populations, and the fourth only in the Togiak population. HRMA patterns were scored as biallelic loci, so even with multiple SNPs only a single polymorphism was considered, resulting in effective binning of rare alleles and conformance to HWE.

The distribution of genetic diversity within and between populations was remarkably similar for SNPs as for previously analyzed markers (allozymes, mtDNA and microsatellites, Tables 5 and 6). Almost all the genetic differentiation was between the two major basins, most likely due to secondary contact of western and eastern Pacific population groups after the last ice age [21], [22]. Similarly, the absence of stable genetic differentiation between Prince William Sound and Kodiak Island from microsatellites [21] was confirmed by SNPs. These patterns of genetic diversity suggest that the 14 loci surveyed here represent true genetic variation and not artifacts caused by paralogous loci or technical problems.

Genetic diversity was higher in the Gulf of Alaska than the Bering Sea (Tables 4 and 5), again conforming to previously reported results from other markers [21], [22]. However, the difference in genetic diversity was higher in SNPs than in the other markers (as measured by the ratio between heterozygosities in the two basins, Table 5). Average heterozygosity was intermediate in SNPs, therefore providing more opportunity for difference in diversity than at markers with very low (allozymes: *H_e_* = 0.10) or very high (microsatellites: *H_e_* = 0.91) variability. Although the difference in diversity between Bering Sea and the Gulf of Alaska may have been due to ascertainment bias (17 of the 23 sequenced fish came from the Gulf of Alaska), the corresponding results between allozymes, microsatellites and SNPs suggest that differences in demographic history may have caused these genetic patterns, rather than a methodological issue in one of the three markers.

Although genetic patterns in SNPs are similar to microsatellites, the distribution of genetic differentiation among individual loci suggested some selective differentiation. Five of the 14 loci showed very high differentiation (*F_ST_*>0.1) between the two ocean basins (Figure 5), and in four of these five loci genetic variability in the Bering Sea was extremely low (*H_e_*<0.06, Table 4; *Cpa_CYP2J5* the exception). Two of these four loci had three and two SNPs, respectively (*Cpa_ABCG5*, *Cpa_28757*), and thus the differentiation may be due to any one of those SNPs. The fifth locus (*Cpa_CYP2J5*) demonstrated significant deficiencies of heterozygotes in both Gulf of Alaska samples. Despite this high differentiation, the variance component between ocean basins was not significant at any of the loci, likely because of the small number of populations (one in the Bering Sea, two in the Gulf of Alaska) and the consequent low power of permutation approaches randomizing entire samples between groups (Arlequin [23]). Nevertheless, the differentiation at these loci appears real and may either be due the different evolutionary history of the Bering Sea population [22] or due to selection in different environments. Further population genetic analyses are required to address this question in a genome scan approach; our data here provide the needed foundation for development of the necessary marker set.

Another locus showed high and significant differentiation between Kodiak Island and PWS (Figure 5; *Cpa_APOB*), and deficiency of heterozygotes in PWS, which may suggest selection within PWS. That locus is a virus response gene, which corresponds to the notion that infection by viral hemorrhagic septicemia virus may have at least contributed to the delayed recovery of Prince William Sound herring [24], but see [25]. However, data are too preliminary to reach any conclusions here, and additional loci need to be screened. Because of the limited number of markers and the biased selection of SNPs in genes coding for pollution and disease relevant genes, we did not attempt a formal outlier test. Nevertheless, these analyses show general correspondence of genetic patterns from SNPs with those of other genetic markers and suggest the value of expanding analyses to many more of the SNPs discovered in this study.

### Conclusions

Targeted transcriptomic sequencing provides a valuable resource for genetic marker and gene discovery in non-model organisms. As part of one of the first large scale sequencing efforts for a member of the order Clupeiformes, we have characterized over 40,000 contigs and have described a workflow for SNP discovery and validation. Five SNPs supported a between ocean basin differentiation, while one SNP also showed significant differentiation between Prince William Sound and Kodiak Island within the Gulf of Alaska. These loci will provide a better understanding of Pacific herring population structure as well as insight into the dynamics of selection and local adaptation.

## Methods

### Tissue Collection

Liver and testes samples from sexually mature Pacific herring were provided by the Alaska Department of Fish and Game from four locations; three in the Gulf of Alaska (Kodiak Island, Prince William Sound and Sitka Sound) and one in the Bering Sea (Togiak Bay). Six fish each were sampled at Kodiak Island, Prince William Sound, and Togiak Bay, and five fish were sampled from Sitka. Tissue samples were immediately preserved in RNAlater (Ambion).

### Sample Preparation

Individual tissue samples were transferred to TriReagent (Molecular Research Center), and total RNA was isolated as per manufacturer's instruction. Individual liver and testes RNA samples (four fish and three fish per location, respectively) were pooled in equal quantity for the construction of two Pacific herring transcriptome libraries. Messenger RNA was isolated from each total RNA pool with the MicroPoly(A) Purist Kit (Ambion) according to the manufacturer's protocol. The isolated mRNA was purified again with the MicroPoly(A) Purist Kit (Ambion) to further minimize residual rRNA carryover. Libraries were constructed by MOgene, LC (St. Louis, MO), following standard protocols from Roche Life Sciences.

### Sequencing and Analysis

Both libraries were sequenced using the Genome Sequencer FLX System (Roche) at MOgene, LC (St. Louis, MO). Initially, all sequences were trimmed based on quality scores of 0.05 [27], [28] and a maximum allowance of two ambiguous nucleotides. Sequences smaller than 100 bp were removed. *De novo* assembly was carried out using CLC Genomics Workbench v3.7 (CLC Bio) with the following parameters: similarity = 0.98, length fraction = 0.9, insertion cost = 3, deletion cost = 3, mismatch cost = 2 and minimum size = 300. Where reference assemblies were performed, the same parameters were applied. Consensus sequences were compared to the Swiss-Prot database (http://uniprot.org) in order to determine putative annotation. Associated GO terms (Gene Ontology database: http://www.geneontology.org) were used to classify sequences based on biological process as well as categorize genes into parent categories (GO slim).

RNA-seq analysis was used to characterize the transcriptome tissue specificity (CLC Genomics Workbench v3.7 (CLC Bio)). Expression values were measured in RPKM (reads per kilobase of exon model per million mapped reads, see [29]). Parameters for RNA-seq analysis included an unspecific match limit of 10 and a minimum length fraction of 0.9. Given the resources necessary to perform pyrosequencing and the absence of the sequenced genome in herring, we evaluated the relative benefit of additional sequencing effort by rarefaction analysis. Specifically, quality trimmed reads from each library were sequentially sampled in 1×10^5^ sequence read increments and *de novo* assembled as described.

### SNP Discovery

Candidate SNPs were identified from assembled reads using CLC Genomics Workbench v3.7 (CLC Bio). Parameters were as follows: maximum gap and mismatch count = 2, minimum average quality = 15, minimum central quality = 20, minimum coverage = 4, minimum variant frequency (%) = 35.0, window length = 11. In order to determine whether a SNP resulted in a potential amino acid substitution, putative open reading frames were identified and SNP detection carried out as described. Specifically, open reading frames were identified in the consensus sequences from the *de novo* assembly of all reads based on the inclusion of start and stop codons, with a minimum size of 100 bp, using *getorf* (EMBOSS). All quality trimmed reads were then mapped back to these possible open reading frames, and SNP detection was carried out as described (CLC Genomics Workbench v3.7 (CLC Bio)). Consensus sequences generated with *getorf* (EMBOSS) which contained putative SNPs were compared to the Swiss-Prot database.

### SNP Selection and Primer Testing

Ninety-six putative SNPs were selected for validation. Selection stringency was increased from parameters described above to include a window length of 151. In addition, functional annotation was taken into consideration. PCR primers were designed using Primer3 [30] to amplify a template approximately 200 bp long that contained a single putative SNP in the ascertainment fish. A PCR test was done using 2× LightCycler480 High Resolution Melting Master (Roche Applied Science) following manufacturer's instructions. Primer pairs that produced a single, clean amplicon were sequenced. Specifically, six individuals were sequenced in both directions using ABI PRISM BigDye Terminator version 3.1 Cycle Sequencing Kit and analyzed on a 3730 DNA Analyzer (AB) by High-Throughput Sequencing Solutions (University of Washington, Department of Genome Sciences). Sequence chromatograms were aligned and visually screened for polymorphisms using Sequencher 4.9 (GeneCodes Corporation).

### High Resolution Melt Analysis and Genotyping

Those SNPs validated using Sanger sequencing were genotyped using high resolution melt analysis (HRMA) [31], [32]. A total of 95 individuals from each of three populations sampled from Kodiak Island, Prince William Sound, and Togiak Bay, Alaska, were genotyped. Genomic DNA was extracted using DNeasy 96 Blood & Tissue Kits (QIAGEN) and quantified using Quant-iT PicoGreen dsDNA Assay Kit (Invitrogen) following manufacturer's instructions. Fluorescence was measured in a 200 µL reaction on a VICTOR3 multilabel microplate reader (Perkin Elmer). DNA concentrations were then normalized for HRMA. PCR was conducted in a 10 µL volume containing 10 ng of genomic DNA, 1× LightCycler 480 High Resolution Melting Master (Roche Applied Science), 3.5 mM MgCl2, and 0.2 µM each PCR primer. Primers used for HRMA are provided in Table 2. Thermal cycling was performed on a Veriti 384-Well Thermal Cycler (Applied Biosystems; AB) as follows: 95°C hold for 10 min followed by 45 cycles of 95°C for 15 sec, 60°C for 15 sec, and 72°C for 15 sec. The plates were transferred to a LightCycler 480 Real-Time PCR System (Roche Diagnostics) after PCR and heated to 95°C for 1 min, then cooled to 40°C for 1 min. HRMA data were collected between 62°C and 95°C at 25 acquisitions per °1C, using a ramp rate of 0.02°C per second. The amplicons were analyzed for the presence of discrete melt-curve families, signaling the presence of SNPs, using the LightCycler 480 Gene Scanning Software v. 1.5.0 SP1 (Roche Diagnostics). Genotypes were inferred from clearly resolved melt families [8], [32].

### Data Analyses

Deviations from Hardy Weinberg Equilibrium (HWE) and significance of population differentiation were tested using Fisher's exact test in Genepop 4 [33]. A hierarchical locus-by-locus AMOVA was carried out in Arlequin v 3.5 [23], with ocean basins (Gulf of Alaska, Bering Sea) as groups.

## Supporting Information

Table S1 **Description of select contig sequences.** Description is based on BlastX analysis using the Swiss-Prot database. E-values and organism associated with BlastX hits are provided as are the number of unique reads that map to each contig.
(XLS)
